# The Pro-children Project- a cross-national approach to increasing fruits and vegetables in the next generation and onwards

**DOI:** 10.1186/1479-5868-3-26

**Published:** 2006-09-14

**Authors:** Annie S Anderson

**Affiliations:** 1 Centre for Public Health Nutrition Research, Department of Medicine, University of Dundee, Ninewells Hospital and Medical School, Dundee, DD1 9SY, Scotland, UK

## Abstract

This editorial introduces the special series in the International Journal of Behavioral Nutrition and Physical Activity on the Pro Children study (Promoting and Sustaining Health through Increased vegetable and Fruit Consumption among European Schoolchildren). The Pro Children study is a new and innovative study that takes a cross -national approach to identifying the factors associated with fruit and vegetables consumption in European children (from nine countries) and evaluating a multi-centre intervention programme. A full set of papers on many aspects of the study will appear as a series in the IJBPNA allowing a more detailed view of design, behavioural theoretical constructs, action, methods for implementation, assessment tools, impacts and outcomes to be reported. Areas for future work will also be identified and discussed in the context of the 21st century developed world environment which is superb at encouraging the promotion of energy dense foods and drinks.

## Background

The challenge of increasing intakes of fruits and vegetables in children and adolescents must be one the worthiest nutrition interventions to tackle in the list of possible routes to improving health. Much time, money and effort has been spent in developing a magic formula to motivate, coerce and enthral youth into chomping their way to health through a cocktail of exciting and powerful phyto-chemicals but we still have much to learn about effective approaches

Developing, implementing and evaluating dietary interventions for children offers many opportunities for testing behavioural theories, examining the power of different settings and examining the impact by a range of different socio-economic characteristics. Interventions to increase intakes of fruits and vegetables have of course been widely reported but often these have been of short duration [1] culturally relevant to specific geographic regions [2] or inadequately evaluated [e.g. for nutrient implications] [3].

The Pro-children Project [4] is a new and innovative study that takes a cross -national approach to identifying the factors associated with fruit and vegetables consumption in European children [from nine countries] and evaluating a multi-centre intervention programme. A full set of papers on many aspects of the study will appear as a series in the IJBPNA allowing a more detailed view of design, behavioural theoretical constructs, action, methods for implementation, assessment tools, impacts and outcomes to be reported. Areas for future work will also be identified and discussed in the context of the 21^st ^century developed world environment, which is superb at encouraging the promotion of energy dense foods and drinks.

There are few large-scale interventions that have been systematically designed to inform policy makers about cost effective and clinically relevant approaches to assisting children eat more fruits and vegetables. The Pro-children Project will make a valuable contribution to the evidence base about dietary outcomes, changes in psychosocial variables and sustainability. Issues of school and community policy and availability of fruits and vegetables deserve to be widely disseminated to the scientific community, professional organisations, policy makers and to the general public.

In this issue of the journal, the study team presents a systematic review of the determinants of fruit and vegetable consumption among children and adolescents. The findings confirm the large amount of research that has been undertaken on this dietary challenge but also flag problems of representativness of population sub groups, failure to use valid and reliable data collection tools and lack of detailed analytical approaches. This overview provides a good canvass of the landscape for growing and designing the Pro-Children Study. Further papers will present details of protocols, actions and effects providing further food for thought for future intervention work.
